# Renal Denervation for Treating Resistant Hypertension: Current Evidence and Future Insights from a Global Perspective

**DOI:** 10.1155/2013/513214

**Published:** 2013-11-27

**Authors:** Y. Castro Torres, Richard E. Katholi

**Affiliations:** ^1^Facultad de Medicina, Universidad de Ciencias Médicas “Dr. Serafín Ruiz de Zárate Ruiz”, Santa Clara, 50100 Villa Clara, Cuba; ^2^Southern Illinois University School of Medicine and Prairie Education and Research Cooperative, Springfield, IL 62701, USA

## Abstract

Adequate blood pressure control represents an important goal for all physicians due to the complications of hypertension which reduce patients' quality of life. A new interventional strategy to reduce blood pressure has been developed for patients with resistant hypertension. Catheter-based renal denervation has demonstrated excellent results in recent investigations associated with few side effects. With the growing diffusion of this technique worldwide, some medical societies have published consensus statements to guide physicians how to best apply this procedure. Questions remain to be answered such as the long-term durability of renal denervation, the efficacy in patients with other sympathetically mediated diseases, and whether renal denervation would benefit patients with stage 1 hypertension.

## 1. Introduction

Approximately 34% of adults worldwide have hypertension [1]. Hypertension is the leading cause of global mortality accounting for 13% of deaths [2]. In the United States, there is an estimated of 77.9 million adults ≥20 years of age with this condition [3]. Hypertension management utilizes the strategies of lifestyle modification and pharmacological treatment. However, in many patients, blood pressure (BP) control is not accomplished.

Resistant hypertension (RH) is defined when a patient taking three or more antihypertensive drugs, including a diuretic, at optimal tolerated doses, and still maintains BP values >140/90 mmHg [4, 5]. Prevalence of RH is not well established but some statistics reveal that it represents 13% of hypertensive patients [6]. Data from National Health and Nutrition Examination Survey among United States adults reveal the criteria for RH which were found in 8.9% of hypertensive patients [3]. The causes of RH are diverse and it may involve multiple mechanisms (see Table 1) [4, 5]. RH worsens the prognosis of hypertensive patients. The rate of fatal and nonfatal cardiovascular events in patients with RH is three to six times higher than that of controlled hypertensive individuals. RH increases the risk of left ventricular hypertrophy, microalbuminuria, kidney failure, endothelial dysfunction, carotid artery stiffness, and atherosclerosis [7].

Recently, a catheter-based technique using radiofrequency to destroy the renal nerves has opened a new novel approach to treat RH [8–11]. Clinical trials have shown a reduction in BP with minimal side effects [12, 13]. In this paper we propose to review the current evidence and status of catheter-based renal denervation (RDN) in the treatment of RH and its future as a therapy from a global perspective.

## 2. Renal Sympathetic Nerves

**Figure 1:**
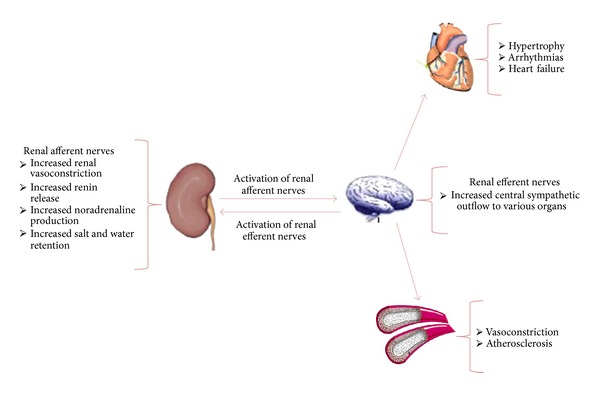
Activation of renal nerves and their actions in different organs. Stimulation of the renal sympathetic efferent nerves causes renin release, sodium retention, and reduced renal blood flow, factors which cause hypertension. Elevated afferent renal sensory nerve signals are centrally integrated in the hypothalamic region and result in increased sympathetic outflow directed to various regions, including the kidneys, the skeletal muscle vasculature, and the heart, which contributes substantially to elevated peripheral vascular resistance, vascular remodeling, and left ventricular hypertrophy.

The pathogenesis of essential hypertension is multifactorial. However, hyperactivity of the sympathetic nervous system plays an important role in its development and progression [14]. The kidneys have an important interrelationship with the sympathetic nervous system. As shown in Figure 1, there are two types of renal sympathetic nerve fibers: the afferent and efferent nerves are located immediately adjacent to the wall of the renal artery. The afferent renal sympathetic nerves have cell bodies located in the ipsilateral dorsal root ganglia and modulate central sympathetic outflow by sending sensorial information from chemoreceptors and mechanoreceptors in the renal tissue. Renal injuries including ischemia and hypoxia increase afferent renal sympathetic nerves activity resulting in increased peripheral sympathetic nerve activity with resultant arterial vasoconstriction and subsequent hypertension.

The efferent renal sympathetic nerves originate from postganglionic sympathetic neurons and transmit signals from the central sympathetic nervous system to the renal vasculature, tubules, and juxtaglomerular apparatus. Efferent renal sympathetic activity is moderated by renorenal reflexes and central sympathetic nervous system outflow. Elevated renal efferent nerve activity increases sodium reabsorption, increases renin release, and causes renal arterial vasoconstriction, factors which cause hypertension [15–18].

## 3. Renal Denervation

The importance of the renal nerves in treating hypertension has evolved over the years. Nephrectomy in humans resulted in a lowering of blood pressure and a reduction of muscle sympathetic nerve activity. Other studies confirmed this hypothesis through surgical denervation [15]. The use of radical surgical sympathectomy and therapeutic splanchnicectomy was abandoned in the 1960s because these methods were associated with severe side effects such as postural hypotension, syncope, impotence, and mobility disturbance. Another factor which led to abandoning these surgical approaches was the development of modern pharmacological antihypertensive medications [8, 19, 20].

A new approach to disrupt renal sympathetic nerves has been developed without affecting other abdominal, pelvic, or lower extremities innervations. This involves ablation of both afferent and efferent renal sympathetic nerves with a radiofrequency-emitting catheter inserted percutaneously into the renal artery via transfemoral approach to reduce BP in patients with RH. The Symplicity catheter system is now available in many countries. This device consists of a 5-F blind-ending catheter that houses a flexible radiofrequency wire. The back end portion of the catheter handle connects to a radiofrequency generator that supplies power. The procedure is performed under local anesthesia and sedation. Catheter control by the handle allows bringing this device into contact with the endothelial surface of the artery. Four to six 2-minute treatments are delivered at different locations longitudinally and rotationally in order to achieve a helical pattern of ablation within each renal artery. The energy delivered is 5 W to 8 W. Both renal arteries are treated on the same day. Before this procedure, the patient receives anticoagulation and intravenous administration of 200 *μ*g of nitroglycerin [12, 21].

## 4. Evidence in Favor of Catheter-Based RDN

There are several studies in patients with RH which have shown a reduction in BP and minimal side effects with RDN. Symplicity HTN-1 and HTN-2 trials provide much information and are compared in Table 2. Symplicity HTN-1 initially was conducted in 45 patients with RH. The mean values of baseline BP were 177/101 mmHg. In the first six months after RDN, there was a mean decrease in systolic and diastolic BP of 25 mmHg and 11 mmHg, respectively. When the sample size was increased to 153 patients, the mean decrease in systolic and diastolic BP was 32 mmHg and 14 mmHg, respectively. In 10 of the patients, a 47% reduction in renal norepinephrine spillover one month after RDN was measured confirming renal efferent nerve attenuation. The side effects of RDN were one case of artery dissection and another with a prior renal artery stenosis. A small number of patients and no control group were the main limitations of this study [12].

Symplicity HTN-2 was the next study performed to determine the utility of the catheter-based RDN to reduce BP in patients with RH. It was an international multicenter randomized trial. A total of 106 patients with RH and preserved renal function were enrolled. In this study, the participants were divided into 2 groups. The first group (*n* = 52) was randomly assigned to undergo RDN with the Symplicity device. The second (*n* = 54) continued with antihypertensive therapy alone. The mean baseline values in both groups were 178/97 and 178/98 mmHg, respectively. The results showed that the RDN group had a mean office BP reduction of 20/7 and 32/12 mmHg by 1 and 6 months. Nineteen of these patients had a reduction in systolic BP to less than 140 mmHg. Adverse events included had one case of postprocedural hypotension which required a reduction in antihypertensive drugs, a single femoral artery pseudoaneurysm, and 7 patients required atropine due to intraprocedural bradycardia. Renal function showed no significant differences between groups at six months [13].

One-year results have been published of the patients enrolled in Symplicity HTN-2. The mean fall in systolic BP at 12 months after the procedure was 28 mmHg. Knowing the long-term benefit of this procedure will determine its application in clinical practice [22].

A number of isolated cases have reported the utility of RDN. Ong et al. published a case report of a 76-year-old male subject diagnosed with RH treated with percutaneous RDN with resultant marked lowering of BP by six months. It was possible to reduce antihypertensive drugs from five to one in the patient [23]. Similar findings were found with RDN in a 59-year-old male with longstanding RH and 2 previous episodes of transient ischemic attacks. The researchers observed a reduction in serum noradrenaline levels and plasma renin activity with an improvement in baroreflex sensitivity [21].

Another prospective study was carried out in China with the aim to evaluate the safety and short-term efficacy of RDN therapy in 8 patients (6 males and 2 females). One month and three months after RDN, mean systolic and diastolic BP decreased significantly compared with baseline. There were no complications or significant changes in renal function [24]. The utility of RDN has been shown in other settings. In order to determine the effects of this procedure on central hemodynamics in patients with RH, RDN was performed in 110 patients and 10 controls. The main findings were a significant reduction in central aortic BP in the RDN group from 167/92 mmHg to 149/88 mmHg, 147/85 mmHg, and 141/85 mmHg at 1, 3, and 6 months (*P* < 0.001), respectively. Aortic pulse pressure decreased from 76.2 ± 23.3 mmHg to 61.5 ± 17.5 mmHg, 62.7 ± 18.1 mmHg, and 54.5 ± 15.7 mmHg 1, 3, and 6 months after RDN (*P* < 0.001), respectively. This investigation was the first to show that RDN can improve central hemodynamics. This study also reported significant improvement in arterial stiffness in patients treated with RDN [25].

Variability of BP is an independent factor that contributes to organ damage. Previously, it has been demonstrated that an increase in sympathetic nervous system activity is linked with BP variability. Zuern et al. conducted an investigation which enrolled 11 patients with RH. The mean age was 68.9 ± 7.0 years, baseline systolic BP was 189 ± 23 mmHg, and patients were on 5.6 ± 2.1 antihypertensive drugs. Six months after RDN, a reduction in BP variability was observed. Previously, a reduction in BP variability has only been demonstrated with the administration of calcium antagonist drugs. This study demonstrated a reduction in BP variability with RDN. The authors suggest that identification of patients with elevated BP variability may be useful when selecting patients who would benefit from RDN. Patients with elevated BP variability are at increased risk for stroke and transient ischemic attacks. Thus, RDN in these patients might reduce the incidence of these conditions [26].

Recently, the effects of RDN were assessed in 10 patients with severe hypertension and sleep apnea. After RDN, systolic and diastolic BP were decreased by 34/13 mmHg at 3 and 6 months. Also, a decrease in apnea-hypopnea index and significant decreases in plasma glucose concentration and glycated hemoglobin levels at 6 months were observed [27]. This study suggests that a reduction in sympathetic nervous system activity by RDN reduces serum glucose levels possibly by decreasing insulin resistance. The benefit of RDN in type 2 diabetic patients with hypertension merits further study. This investigation also suggests that patients with sleep apnea intolerant to continuous positive airway pressure are a subgroup which could benefit from RDN.

## 5. Eligible Patients for RDN

Based on the results from catheter-based RDN to reduce BP in patients with RH, some medical societies have developed guidelines for physicians and interventional practitioners on the proper indications for this technique. Although this approach is new, its availability has grown quickly around the world. Evaluation of benefits/risk ratio with this procedure was the main element taken into account to develop these guidelines or consensus. In 2012, the French Societies of Hypertension, Cardiology and Radiology proposed to limit RDN to patients with essential hypertension uncontrolled by four or more antihypertensive drugs; one of these drugs should be a diuretic or spironolactone. The patients should have a measurement of office blood BP over 160 and/or 100 mmHg confirmed by ambulatory BP measurement. The ambulatory BP should be more than 135/85 mmHg during the daytime period. These experts also recommended monitoring of BP, renal function, and anatomy of renal arteries 12 months and 36 months after procedure. It was emphasized that pharmacological treatment of hypertension should not be interrupted after RDN because the BP often decreases slowly over time after RDN. Since RDN is a complex procedure, it should be performed by interventionists with experience in this field [28].

The European Society of Cardiology also published a consensus statement regarding the use of catheter-based RDN for the treatment of hypertension [29]. Patients eligible to receive this procedure should meet the following criteria: office-based BP ≥ 160 mmHg (≥150 mmHg in patients with type 2 diabetes), three or more antihypertensive drugs in adequate, including use of a diuretic, having attempted to modify BP with lifestyle changes, secondary hypertension having been excluded, pseudoresistance having been excluded with the use of ambulatory blood-pressure monitoring (ABPM), patients having preserved renal function (glomerular filtration rate ≥ 45 mL/min/1.73^2^), absence of polar or accessory arteries, no renal artery stenosis, and no prior renal revascularization.

In summary, hypertensive patients being considered for RDN should not be considered to be resistant unless pseudoresistance has been excluded with the use of ABPM and they have been evaluated for sleep apnea and secondary causes of hypertension.

## 6. Unanswered Questions

Although investigations have demonstrated the efficacy of RDN to decrease BP in patients with RH, there are still unanswered questions which should be addressed in order to increase our knowledge about this topic and modify future guidelines. It is important to determine the durability of RDN. Available data support the efficacy of this procedure to at least 2 years [30]. Further followup will answer whether this technique is effective after this time. However, a growing number of recent studies have shown that RDN has little blood pressure lowering effect in many patients and that there is no reliable method to predict success or failure in the clinical setting. Developing methods to select RH patients with hyperactivity of the sympathetic nervous system who would likely respond to RDN merits further investigations. Also, it still has not been established if there is a correlation between lower BP after RDN and an improvement in cardiovascular outcomes in patients with heart failure, coronary artery disease, and other conditions which are worsened by hypertension.

## 7. Perspectives

Many patients could benefit from RDN. A concern about using ABPM in selecting RH patients is the additional cost of this procedure. In a recent work, Geisler et al. found that RDN can reduce the risk of stroke, myocardial infarction, coronary heart disease, heart failure, and end-stage renal disease in a state-transition model. They also established that over a wide range of assumptions, RDN is a cost-effective strategy for RH. However, the costs of RDN are $12,500 (one-time material and procedure cost; $8,000 to $15,000) [31]. This limits the ability to offer this procedure to patients in underdeveloped countries. Another study supports the idea that RDN is a cost-effective intervention for patients with RH. The authors suggest that potential lifetime cost-effectiveness ratios may be increased when the procedure is performed earlier in RH patients [32].

Recently, 46 patients underwent RDN to investigate the effect on BP, on left ventricular hypertrophy, and systolic and diastolic functions in patients with RH. Besides reduction of systolic and diastolic BP (22.5/7.2 mmHg at 1 month and 27.8/8.8 mmHg at 6 months, *P* < 0.001 at each time point), RDN significantly reduced mean interventricular septum thickness from 14.1 ± 1.9 mm to 13.4 ± 2.1 mm and 12.5 ± 1.4 mm (*P* < 0.007). Ejection fraction significantly increased after RDN (baseline: 63.1 ± 8.1% versus 70.1 ± 11.5% at 6 months, *P* < 0.001). In 18 matched control patients, there were no significant changes [33]. This study suggests that RDN facilitates regression of left ventricular hypertrophy in patients with RH. Since patients with heart failure often have hyperactivity of the sympathetic nervous system, RDN may reduce future risks of these patients.

Clinical trial data from Symplicity radiofrequency catheter systems have created much interest in the role of the renal nerves in hypertension and other cardiovascular conditions. Furthermore, the attenuation of BP observed has led to the rapid development of alternative methods of RDN by radiofrequency ablation as well as by ultrasound ablation and perivascular pharmacologic ablation [34, 35]. Many clinical trials investigating these various innovative approaches to achieve RDN are ongoing.

## 8. Conclusions

Catheter-based RDN constitutes a novel treatment for RH. The two seminal studies which have evaluated this procedure in RH patients have demonstrated its safety and efficacy. Hypertensive patients being considered for RDN should not be considered to be resistant unless pseudoresistance has been excluded with the use of ABPM and secondary causes of hypertension have been ruled out. Further investigations and followup are needed to determine the long-term durability of RDN, the possible modification of circadian variation of BP after RDN, its efficacy in other diseases such as heart failure, stroke, and kidney failure, and its use in stage 1 hypertension. Currently, there are no clinical trial data available to indicate that RDN improves cardiovascular outcomes.

## Figures and Tables

**Table 1:** Causes of RH.

Improper blood pressure measurement	Associated conditions
Volume overload and pseudotolerance	(i) Obesity
(i) Excess sodium intake	(ii) Excess alcohol intake
(ii) Volume retention from kidney disease	(iii) Physical inactivity
	(iv) Low-fiber diet

Drug-induced or other causes	Secondary causes of RH

(i) Nonadherence	(i) Obstructive sleep apnea
(ii) Inadequate doses	(ii) Primary aldosteronism
(iii) Inappropriate combinations	(iii) Pheochromocytoma
(iv) Cyclosporine and tacrolimus	(iv) Hyperparathyroidism
(v) Cocaine, amphetamines, and other illicit drugs	(v) Aortic coarctation
(vi) Sympathomimetics (decongestants, anorectics)	(vi) Renal parenchymal disease
(vii) Herbal compounds	(vii) Renal artery stenosis
(viii) Adrenal steroids	(viii) Intracranial tumor
(ix) Nonsteroidal anti-inflammatory drugs; aspirin, cyclooxygenase 2 inhibitors	
(x) Erythropoietin	
(xi) Licorice (including some chewing tobacco)	
(xii) Oral contraceptives	

**Table 2:** Comparison between Symplicity HTN-1 and HTN-2 trials.

Trial	Patients characteristics	Study design	Mean values of baseline BP	Main results	Side effects
Symplicity HTN-1	45 patients, mean age 58 ± 9 years	Proof-of-principle, nonrandomized	177/101 ± 20/15 mmHg	At 6 months showed a mean decrease in systolic and diastolic BP of 25 mmHg and 11 mmHg respectively. Reduction in renal norepinephrine production by 47% in 10 patients	The side effects were one case of renal artery dissection and another with a prior renal artery stenosis.

Symplicity HTN-2	106 (52 RD, 54 controls). Mean age 58 ± 12 years in both groups	Randomized, controlled; unblinded	RD group: 178/97 ± 18/16 mmHg Control group: 178/98 ± 16/17 mmHg	RD group had mean office BP reduction of 20/7 and 32/12 mmHg by 1 and 6 months following RD. Nineteen patients had a reduction in systolic BP to less than 140 mmHg in this same group	One case of postprocedural hypotension. A patient with single femoral artery pseudoaneurysm and 7 patients developed intraprocedural bradycardia
